# Serum neurofilament light chain and postural instability/gait difficulty (PIGD) subtypes of Parkinson’s disease in the MARK-PD study

**DOI:** 10.1007/s00702-022-02464-x

**Published:** 2022-01-24

**Authors:** Monika Pötter-Nerger, Janina Dutke, Susanne Lezius, Carsten Buhmann, Robert Schulz, Christian Gerloff, Jens Kuhle, Chi-un Choe

**Affiliations:** 1 Department of Neurology, University Medical Center Hamburg-Eppendorf, Martinistraße 52, 20246 Hamburg, Germany; 2 Institute of Biometry and Medical Epidemiology, University Medical Center Hamburg-Eppendorf, Hamburg, Germany; 3 Department of Neurology, University of Basel, Basel, Switzerland

**Keywords:** MDS-UPDRS, Hoehn and Yahr, MoCA, Biomarker

## Abstract

The PIGD (postural instability / gait difficulty) subtype of Parkinson´s disease (PD) is associated with faster cognitive and motor decline. So far, there are no quantifiable biomarkers to aid clinical subtyping. Neurofilament light chain (NfL) is a highly specific marker of neuro-axonal damage and can be assessed in blood. Here, we investigated if serum NfL concentrations are associated with PIGD subtype and PIGD scores in PD patients at advanced disease stages. Furthermore, we evaluated if serum NfL is associated with motor and cognitive function assessed with MDS-UPDRS part III and Montreal cognitive assessment (MoCA). Serum NfL levels were analyzed with Single Molecule Assays (Simoa) in blood of 223 PD patients from the *bioMARKers in Parkinson’s Disease* (MARK-PD) study. Serum NfL concentrations were higher in PIGD patients independent of age, sex and disease duration. In linear regression analysis, serum NfL levels were associated with MoCA, MDS-UPDRS III and PIGD scores in unadjusted models, but remained significant after adjustment only with PIGD scores. In conclusion, increased serum NfL levels were associated with PIGD subtype and PIGD scores in patients with advanced PD.

**Supplementary Information:**

The online version contains supplementary material available at 10.1007/s00702-022-02464-x.

## Introduction

Postural and gait disturbances represent therapeutically demanding symptoms with high impact on quality of life in Parkinson´s disease (PD). These axial symptoms are key features for one of the most used definitions of clinical motor subtypes of PD patients, which are classified into “PIGD” (Postural Instability / Gait Difficulty subtype), “TD” (Tremor Dominant subtype) or “Intermediate” subtypes (Jankovic et al. 1990; Stebbins et al. 2013). Cut-off values of the PIGD score, which are based on the MDS-UPDRS part III, define these PD subtypes. The PIGD score is also a continuous marker of axial symptoms and has prognostic and therapeutic implications (van Rooden et al. 2011). PIGD subtype patients exhibit a faster disease progression, a higher risk of cognitive decline, sleep disturbances, fatigue and autonomic dysfunction, an increased risk of freezing and falls, a shorter survival time and a worse quality of life compared with TD patients (Huang et al. 2019; Kwon et al. 2021; Lord et al. 2020; Ren et al. 2020a, b, 2021). Based on this phenomenological, symptom-orientated descriptive classification, motor subtypes change with disease progression and treatment (Luo et al. 2019). Therefore, more stable classification systems are warranted, which are based on the underlying pathophysiological differences and specified by additional biomarkers.

Blood-based biomarkers are easily accessible, quantifiable, objective parameters and can reflect the involved pathophysiological processes. Among these biomarkers, blood neurofilament light chain (NfL) has the potential to predict disease severity and duration as well as motor and cognitive decline (Hansson et al. 2017; Khalil et al. 2018; Lin et al. 2019; Mollenhauer et al. 2020; Niemann et al. 2021). Blood NfL levels reflect the degree of neuroaxonal damage. In early PD, NfL levels were higher in PIGD compared with TD patients after a 2 year follow-up, but not at baseline (Ng et al. 2020). Furthermore, higher NfL levels were associated with a worse cognitive and motor function and decline during follow-up at early stages (Ng et al. 2020). However, the link between blood NfL with PIGD subtype and score in patients with advanced PD is unknown. Another recent study with 376 de novo PD patients from the PPMI database confirmed that higher serum NfL at baseline were associated with intense worsening of MDS-UPDRS III and PIGD scores, but not with progression of MoCA scores (Ye et al. 2021).

Here, we evaluated if serum NfL levels were associated with PIGD subtype and PIGD scores in advanced PD patients.

## Methods

### Study design, ethical approval and patient consent

The *bioMARKers in Parkinson’s disease* (Mark-PD) study is a prospective observational single-center biobank at the University Medical Center Hamburg-Eppendorf, whose details have been described previously (Choe et al. 2020). The inclusion criteria were as follows: age > 18 years, a clinical diagnosis of Parkinson’s disease fulfilling Queen Square Brain Bank criteria. For this study, 223 PD patients were included with available clinical data to calculate PIGD scores and available serum NfL concentrations. The study protocol was approved by the Ethics Committee of the Hamburg Board of Physicians (PV5298). The investigation was conducted in accordance with the Declaration of Helsinki. Written informed consent was obtained from all participants.

### Clinical assessment

Clinical assessments were performed as previously described (Choe et al. 2020). For this cross-sectional analysis, we used clinical assessments at baseline. Motor impairment was assessed using the Movement Disorder Society Unified Parkinson’s disease Rating Scale part 3 (MDS-UPDRS III) in the ON medication and in case of deep brain stimulation ON stimulation state. Montreal cognitive assessment (MoCA) was used to assess cognitive function. H&Y stage was recorded from medical records. Levodopa equivalent daily dose (LEDD) was calculated. Past medical history, including comorbidities (arterial hypertension, hypercholesterinemia, diabetes mellitus, prior stroke, prior myocardial infarction, heart failure, atrial fibrillation) and laboratory parameters (GFR) were documented from medical records. Dysautonomia was defined as diagnosis of orthostatic dysregulation, pathological Schellong-test or pathological MIGB-SPECT, as previously described (Choe et al. 2020). We calculated from the MDS-UPDRS the ratio of the tremor sum score (part II item 2.10, part III item 3.15–18) and the PIGD sum score (part II item 2.12, 2.13, Part III 3.10–3.12) (Stebbins et al. 2013). Accordingly, TD/PIGD scores ≤ 0.90 classified PIGD subtypes, whereas TD/PIGD scores > 0.90 were defined as non-PIGD. Motor and cognitive decline were defined as an increase of more than 4 points in the MDS-UPDRS III and more than 2 points in the MoCA score during the follow-up period (Schwedhelm et al. 2021).

### Laboratory analysis

The laboratory measurements were obtained from blood samples collected at baseline and processed as previously described (Choe et al. 2020). After centrifugation, serum samples were frozen at − 80 °C until biomarker analyses. Serum NfL levels were determined by single molecule array (Simoa) assay using the capture monoclonal antibody (mAB) 47:3 (initial dilution 0.3 mg/mL; article number 27016) and the biotinylated detector mAB 2:1 (0.1 μg/mL; article number 27018) from UmanDiagnostic transferred onto the Simoa platform, as previously described (Niemann et al. 2021). The samples from the same participants were analyzed together in the same run to avoid within-subjects run-to-run variability. Intra- and interassay variability of the measurements were evaluated with three native serum samples in five consecutive runs on independent days. The mean coefficients of variation (CVs) of duplicate determinations for concentration were 8.5% (9.5 pg mL − 1, sample 1), 5.4% (23.2 pg mL − 1, sample 2) and 7.8% (98.5 pg mL − 1, sample 3). Interassay CVs for serum were 7.8% (sample 1), 8.3% (sample 2) and 4.9% (sample 3).

### Statistical analysis

Continuous variables are given as mean ± standard deviation (SD) if normally distributed, otherwise as median [25th−75th percentile], and categorical variables are given as numbers (percentage) of participants. Relationships between continuous variables were assessed by Spearman’s rank correlation (r). For linear regression analysis, NfL levels were log2-transformed. The association of continuous parameters with PD subtype (PIGD or non-PIGD) was assessed by linear regression models depicted by beta coefficients with corresponding 95% confidence interval (CI) and *P*- value are given. Linear regression analyses were performed unadjusted (model 1) and adjusted for age, sex and disease duration (model 2). *P* values for comparison of 2 groups were obtained by Mann–Whitney-*U*-test for continuous and *X*^2^ test for categorical variables, as appropriate.

Time to motor or cognitive decline was compared by log rank test and Kaplan–Meier curves were used for illustration. The following Cox Proportional Hazards models were used to examine the dependence of motor or cognitive decline: unadjusted (model 1) and adjusted for age, sex, disease duration and baseline MDS-UPDRS III or MoCA score, respectively (model 2). Presented are Hazard Ratios (HR) with corresponding 95% CI and *P* values.

All tests were two-sided and a *P* value < 0.05 was considered statistically significant. *P* values were not adjusted for multiplicity due to the explorative character of the study. Statistical analysis was performed with IBM SPSS Statistics (version 27, IBM Corp., Armonk, NY).

## Results

### Patient characteristics

In this Mark-PD sub-cohort, 223 PD patients were included with an average age of 65.5 ± 9.3 years, a disease duration of 11 [6.5, 15.5] years and 65% male (Table 1). Median NfL concentrations were 18.1 [12.6, 27.2] (Table 1). Among these patients, 73% were classified as PIGD subtypes according to PIGD scores. The median LEDD was 901 [640, 1263] mg/d, MDS-UPDRS III score was 23 [17, 31] and Hoehn and Yahr stage was 2 [2, 3].

**Table 1:** Baseline characteristics of non-PIGD and PIGD subtypes in Mark-PD

Characteristics	non-PIGD (*n* = 61)	PIGD (*n* = 162)	*P* value	Mark-PD subcohort (*n* = 223)
Demographic parameters				
Age, years	63.3 (9.8)	66.4 (9.0)	0.025*	65.5 (9.3)
Male	45 (73)	101 (62)	0.110	146 (65)
Dysautonomia	16 (26)	39 (24)	0.739	55 (25)
Hypertension	29 (48)	61 (38)	0.180	90 (40)
Hyperlipidemia	9 (15)	18 (11)	0.457	27 (12)
Diabetes	9 (15)	13 (8)	0.133	22 (10)
Prior myocardial infarct	6 (10)	13 (8)	0.666	19 (9)
Prior stroke	3 (5)	9 (6)	0.851	12 (5)
Congestive heart failure	4 (7)	5 (3)	0.240	9 (4)
Atrial fibrillation	3 (5)	13 (8)	0.423	16 (7)
Laboratory parameters				
CRP, mg/dl	0.53 [1.10, 2.55]	1.33 [0.62, 2.94]	0.186	1.2 [0.6, 2.9]
Creatinine, mg/dl	0.81 [0.71, 0.89]	0.77 [0.67, 0.89]	0.121	0.78 [0.68, 0.88]
Nfl, pg/ml	15.5 [9.8, 22.1]	19.2 [13.9, 33.2]	< 0.001***	18.1 [12.6, 27.2]
Neurological parameters				
MDS-UPDRS III, points	23 [17, 30]	23 [16, 32]	0.830	23 [17, 31]
Hoehn and Yahr, stage	2 [2, 2.5]	2 [2, 3]	0.290	2 [2, 3]
Motor fluctuations	30 (50)	111 (69)	0.011*	141
LEDD, mg/d	672 [430, 938]	1005 [734, 1379]	< 0.001***	901 [640, 1263]
MoCA, points	27 [25, 29]	26 [23, 28]	0.001**	27 [24, 28]
Disease duration, years	8.5 [4, 13]	12 [8, 17]	0.001**	11 [6.5, 15.5]
DBS	14 (23)	63 (39)	0.026*	77 (35)

Data are mean (SD), *n* (%) or median [IQR], as appropriate. Categorical variables are given as numbers (percentages) of participants. Comparisons between non-PIGD and PIGD subtypes were analyzed with *t* test, Mann–Whitney test or Chi squared test, as appropriate (*P < 0.05, **P < 0.01, ***P < 0.001).

*CRP* C-reactive protein; *Nfl* neurofilament light chain; *UPDRS* unified Parkinson’s disease ranking scale; *LEDD* L-dopa equivalence dose; *MoCA* Montreal cognitive assessment; *DBS* deep brain stimulation *PIGD* postural instability / gait difficulty

### Clinical and laboratory parameters of PIGD and non-PIGD patients

Compared with non-PIGD patients, PIGD patients had a longer disease duration, higher LEDD, lower MoCA scores, more often motor fluctuations and DBS treatment (Table 1). Among laboratory parameters, PIGD patients had increased serum NfL concentrations. In unadjusted and adjusted regression models, differences between non-PIGD and PIGD patients remained significant for MoCA score and serum NfL, whereas MDS-UPDRS III scores did not significantly differ between PIGD and non-PIGD patients (Table 2). Similar results were obtained after excluding PD patients with DBS (Supplementary Table 2).

**Table 2:** Linear regression analysis of PIGD subtype with MDS-UPDRS III, MoCA and serum NfL

	Model	MDS-UPDRS III		MoCA		NfL	
		Mean difference (95% CI)	*P* value	Mean difference (95% CI)	*P* value	Mean factor (95% CI)	*P* value
PIGD vs non-PIGD	1	0.54 (− 3.01, 4.08)	0.766	− 1.98 (− 3.04, − 0.90)	< 0.001***	1.40 (1.16, 1.68)	< 0.001***
	2	− 0.11 (− 3.69, 3.47)	0.951	− 1.43 (− 2.45, − 0.41)	0.006**	1.26 (1.07, 1.48)	0.006**

ANCOVA with β coefficients and 95% confidence interval (model 1: unadjusted; model 2: adjusted for age, sex and disease duration)

### Association of serum NfL with MoCA, MDS-UPDRS III and PIGD scores

Besides differentiation of non-PIGD and PIGD patients, we evaluated the continuous associations of serum NfL with clinical and laboratory parameters. In correlation analysis, serum NfL revealed significant relationships with age, MDS-UPDRS III, LEDD, MoCA and PIGD score (Supplementary Table 1). Although serum NfL was significantly associated with MoCA and MDS-UPDRS III scores in unadjusted linear regression analysis, these associations did not remain significant after adjustment for age, sex and disease duration (Table 3). In contrast, serum NfL concentrations were significantly associated with PIGD score in unadjusted regression models and remained significant after adjustment (Table 3). Similar results were obtained after excluding PD patients with DBS, underlining the robustness of this finding (Supplementary Table 3).

**Table 3:** Linear regression analysis of serum NfL with MDS-UPDRS III, MoCA and PIGD score

	Model	MoCA		MDS-UPDRS III		PIGD score	
		Mean difference (95% CI)	*P* value	Mean difference (95% CI)	*P* value	Mean difference (95% CI)	*P* value
NfL (per twofold increase)	1	− 1.19 (− 1.70, − 0.67)	< 0.001***	2.46 (0.77, 4.14)	0.004**	0.25 (0.14, 0.36)	< 0.001***
	2	− 0.45 (− 1.03, 0.13)	0.129	1.25 (− 0.74, 3.25)	0.217	0.15 (0.02, 0.28)	0.022*

Linear regression analysis with β coefficients and 95% confidence interval (model 1: unadjusted; model 2: adjusted for age, sex and disease duration)

## Discussion

Our main findings were that in PD patients with advanced disease (1) serum NfL levels were increased in PIGD subtype (2) PIGD patients had worse MoCA, but similar MDS-UPDRS III total scores compared with non-PIGD patients and (3) increased serum NfL concentrations were incrementally associated with higher PIGD score.

In PD patients, increased blood NfL levels were correlated with worse motor function in a Taiwanese cohort, but not in a Dutch study (Lin et al. 2019; Oosterveld et al. 2020). Here, we did not observe an association of serum NfL with MDS-UPDRS III scores independent of age, sex and disease duration. However, the PIGD score, which is based on MDS-UPDRS III items focussing on postural instability and gait disturbance, revealed a significant association with serum NfL. This finding is in line with a recent study reporting increased serum NfL levels in PIGD patients at earlier disease stages (Ng et al. 2020). Furthermore, increased NfL levels predicted a more intense worsening of the PIGD, but not tremor score (Ye et al. 2021). These findings suggest that a more pronounced neuro-axonal damage underlies PD symptoms of postural instability / gait difficulty. A faster motor and cognitive decline, which are found in PIGD patients, might therefore be consequences of more severe neurodegenerative processes.

In contrast to motor function, the Taiwanese and Dutch study revealed consistent associations of high blood NfL levels with cognitive impairment (Lin et al. 2019; Oosterveld et al. 2020). Although we also observed a correlation between serum NfL with MoCA scores, this association was not independent of age, sex and disease duration. Compared with the Mini-Mental-State-Examination (MMSE) used in previously published studies, we used the MoCA test, which is known to be more sensitive for cognitive deficits. Therefore, serum NfL might be less sensitive to detect milder cognitive changes. Furthermore, median disease durations were much shorter in the Dutch and Taiwanese study compared with PD patients in Mark-PD. It is therefore possible, that the predictive value of serum NfL changes during the course or disease progression.

Surprisingly, baseline NfL levels did not differ between PIGD and tremor subtypes at early disease stages, whereas increased levels were found in PIGD subtypes after 2 years of follow-up in a PD cohort from Singapore (Ng et al. 2020). In line with these findings, serum NfL levels were significantly higher in PIGD patients at advanced disease stages in our cohort. Although the PIGD subtype is based on a phenomenological classification, neurochemical and neuropathological findings suggest a more severe PD pathology. Lower amyloid-beta 42 (Aβ1-42) and phospho-tau 181 concentrations in the CSF were associated with PIGD subtype indicating a higher degree of neurodegeneration (Kang et al. 2013a, b). Similarly, Lewy bodies and amyloid-beta plaques were more abundant in the cortex of PIGD subtypes suggestive of a more severe pathology (Selikhova et al. 2009). Most importantly, these neurochemical and neuropathological findings correlated with a faster motor decline in PIGD patients. Our study suggests that in PD patients with a median disease duration of 11 years, the extent of neurodegeneration is still significantly different between PIGD and non-PIGD patients. Even more important, incrementally increasing serum NfL levels revealed a robust association with PIGD symptoms and scores, which indicate a continuous spectrum of neurodegeneration. The PIGD subtype is therefore more a continuum than a clear cut entity (Kotagal 2016).

A limitation of our study is the cross-sectional, single-center, hospital-based study design, which does not allow to draw causal relationships. Although we present prospective data on a subset of patients, the size of this sub-cohort is very small and does not allow adjustment of multiple covariates.

In conclusion, we propose that serum NfL remains a valid biomarker to distinguish PIGD subtype in advanced PD patients and allows an incremental quantification of PIGD symptoms.

## Supplementary Information

Below is the link to the electronic supplementary material.

Supplementary file1 (DOCX 21 KB)

## Abbreviations

H&Y: Hoehn and Yahr
LEDD: L-DOPA equivalent daily dose
NfL: Neurofilament light chain
NT-proBNP: N-terminal pro B-type natriuretic peptide
PD: Parkinson’s disease
MOCA: Montreal cognitive assessment
MDS-UPDRS: Movement Disorders Society-Unified Parkinson’s disease rating scale
