# Supplementary material for: Serum neurofilament light chain and postural instability/gait difficulty (PIGD) subtypes of Parkinson’s disease in the MARK-PD study
Source: J Neural Transm (Vienna). 2022 Jan 24;129(3):295–300. doi: 10.1007/s00702-022-02464-x (PMC8930951; doi:10.1007/s00702-022-02464-x)
Supplement: Supplementary file 1 — Supplementary file1 (DOCX 21 KB) [file 702_2022_2464_MOESM1_ESM.docx]

**Supplementary Table 1**

**Correlation analyses of serum NfL with clinical and laboratory parameters in all PD patients**

|  | correlation coefficient r | P value |
| --- | --- | --- |
| Age | 0.586 | <0.001* |
| MDS-UPDRS III | 0.171 | 0.010* |
| H&Y stage | 0.031 | 0.655 |
| LED | 0.154 | 0.022* |
| Disease duration | 0.123 | 0.068 |
| MoCA | -0.346 | <0.001* |
| PIGD score | 0.333 | <0.001* |

Spearman correlation analysis (* P<0.05; *** P<0.001; n=223).

**Supplementary Table 2**

**Linear regression analysis of PIGD subtype with MDS-UPDRS III, MoCA and serum NfL in subcohort of PD patients without DBS**

|  | |  | **MoCA** | | **NfL** | |
| --- | --- | --- | --- | --- | --- | --- |
|  | Model | | mean difference (95% CI) | P value | mean factor (95% CI) | P value |
| PIGD  vs  non-PIGD | 1 | | -2.24  (-3.46, -1.03) | <0.001*** | 1.54  (1.24, 1.90) | <0.001*** |
|  | 2 | | -1.62  (-2.80, -0.44) | 0.008** | 1.29  (1.08, 1.54) | 0.006** |

ANCOVA with β coefficients and 95% confidence interval (model 1: unadjusted; model 2: adjusted for age, sex and disease duration; n=144).

**Supplementary Table 3**

**Linear regression analysis of serum NfL with MDS-UPDRS III, MoCA and PIGD score in subcohort of PD patients without DBS**

|  | |  | **MoCA** | | **MDS-UPDRS III** | | **PIGD score** | |
| --- | --- | --- | --- | --- | --- | --- | --- | --- |
|  | Model | | mean difference  (95% CI) | P value | mean difference  (95% CI) | P value | mean difference  (95% CI) | P value |
| NfL (per 2-fold increase) | 1 | | -1.28  (-1.91, -0.65) | <0.001*** | 3.41  (1.21, 5.60) | 0.003** | 0.33  (0.18, 0.48) | <0.001*** |
|  | 2 | | -0.43  (-1.20, 0.34) | 0.266 | 1.47  (1.31, 4.24) | 0.298 | 0.20  (0.02, 0.38) | 0.027* |

Linear regression analysis with β coefficients and 95% confidence interval (model 1: unadjusted; model 2: adjusted for age, sex and disease duration; n=144)
